# Recent Progress in Polyphenol-Based Hydrogels for Wound Treatment and Monitoring

**DOI:** 10.3390/bios15100657

**Published:** 2025-10-01

**Authors:** Lulu Liu, Wenrui Ma, Junju Wang, Xiang Wang, Shunbo Li

**Affiliations:** 1 College of Chemistry and Chemical Engineering, Chongqing University of Science and Technology, Chongqing 401331, China; liu2021@cqust.edu.cn; 2 Department of Clinical Laboratory Medicine, Southwest Hospital, Third Military Medical University (Army Medical University), Chongqing, 400038, China; wer_ma@tmmu.edu.cn; 3 Key Laboratory of Optoelectronic Technology and Systems (Ministry of Education), College of Optoelectronic Engineering, Chongqing University, Chongqing 400044, China; 4 Novel Materials Laboratory, ShanghaiTech University, Shanghai 201210, China; wangxiang@shanghaitech.edu.cn

**Keywords:** polyphenols, hydrogels, wound treatment, wound monitoring

## Abstract

Hydrogels have received increasing attention in biomedical applications owing to their controllable physical and chemical properties, high biocompatibility, and structural similarity to natural biological tissues. Among them, polyphenol-based hydrogels stand out due to the inherent antibacterial, antioxidant, and anti-inflammatory properties, along with their excellent biocompatibility and functional versatility. These features make them highly promising for advanced wound treatment and monitoring applications. This review highlights recent advances in polyphenol-based hydrogels for wound management and monitoring, with an emphasis on their innovative design and integrated functionality. Firstly, an overview of structure, classification, and biological function of polyphenols is introduced. On this basis, the construction methods, functions, and applications of several representative polyphenol-based hydrogels are discussed. Then, the application of polyphenol-based hydrogels on wound treatment and monitoring is comprehensively summarized. In the end, the recently developed microneedles based on polyphenol hydrogels in combination with artificial intelligence in wound management are also discussed. This review aims to provide valuable insights for advancing polyphenol-based hydrogels, not only in their design, preparation, and application for wound healing and intelligent management but also in their future development potential.

## 1. Introduction

Skin is the largest organ of the human body, which is directly exposed to the external environment [1,2]. It is not only the physical barrier to resist pathogen invasion, but also undertakes complex physiological functions such as perception, temperature regulation, and immune defense [3]. However, the skin is prone to both acute mechanical and thermal injuries and the development of chronic wounds, including diabetic, pressure, and venous ulcers [4]. Wound healing is a complex multistep biological process, and bacterial infection or oxidative stress affects the normal healing process [5]. Chronic wounds, such as diabetic wounds, not only bring immense pain to patients, but also impose a considerable economic burden during medical and healthcare treatment. More seriously, the formation of bacterial biofilms on wounds greatly weakens the effectiveness of antibiotic treatment, which further hinders the healing process of wounds, thus posing enormous pressure on the public health system [6]. Wound dressings are an effective strategy for the treatment of various wounds, which can provide an appropriate microenvironment to promote wound healing [7]. Ideal wound dressings are considered to show appropriate strength to adhere to wounds, wound exudate absorption capabilities, and porous structures for drug release during healing [8]. Traditional wound dressings, including gauze cotton and bandages, are the most widely used wound dressings in clinical practice due to their easy availability, cost-effectiveness, and rapid hemostatic ability [9]. The removal of gauze to observe the healing status can easily cause secondary damage to the wound. Therefore, the development of multi-functional wound dressings with the function of real-time monitoring of wound status is essential to provide significant information for the healing progress, thus enabling timely adjustment of treatment strategy as needed [10].

In recent years, the development of nanotechnology, biomaterial science, and biosensing technology has led to the emergence of “intelligent” functional wound dressings. These wound dressings have the ability to accelerate wound repair and integrate the function of real-time monitoring of physiological parameters of wounds, providing a revolutionary tool for personalized healthcare [11]. Hydrogel has become a potential candidate for advanced wound dressings because of its excellent hydrophilicity, high water content, good biocompatibility, adjustable physicochemical properties, and three-dimensional porous network structure [12,13]. Natural polymers such as chitosan [14], gelatin [15], hyaluronic acid [16], alginate [17], and artificially synthesized polymers such as polyvinyl alcohol [18] and polyacrylamide [19] are widely used to prepare wound dressings. However, wound dressings constructed from a single material generally lack intelligent responsiveness to the wound microenvironment, and have no ability to continuously monitor the physiological status during the healing process. It is particularly important to develop intelligent wound dressings with early diagnosis, real-time monitoring, and on-demand treatment.

Polyphenols, which contain at least one aromatic ring and multiple phenolic hydroxyl groups in their chemical structure, are abundantly present in plants [20]. Polyphenols have been proven to be safe for the human body, and as a result, they have been widely applied in the fields of medicine, food, and cosmetics in recent years. Polyphenol-based hydrogels can interact with diverse molecules via multiple non-covalent and covalent bonds, owing to functional groups such as catechol and pyrogallol. These interactions endow the hydrogels with remarkable adhesion and self-healing properties, which are highly desirable for modern biomedical applications. Such capabilities are typically not found in conventional polypeptide-based or nanomaterial-based hydrogels. In addition, polyphenol-based hydrogels demonstrate remarkable biological activities, including antioxidant, antibacterial, anti-inflammatory, and immunomodulatory effects [21]. Incorporation into hydrogel networks not only retains the intrinsic benefits of polyphenols but also synergistically combines enhanced mechanical properties (such as self-healing and tissue adhesion) with therapeutic bioactivities, thereby enabling applications beyond basic wound dressing [22]. As a result, polyphenol-based hydrogels have rapidly evolved from simple wound dressings to versatile multifunctional platforms. Traditional methods of wound monitoring can be time-consuming and limited by the sophisticated instruments or skilled operators. In recent years, the integration of sensor technology into wound dressings has enabled real-time, wireless, and remote monitoring of healing parameters [11]. Combined with intelligent response and on-demand drug release capabilities, sensor-based wound dressings can provide comprehensive insights into the wound microenvironment so as to achieve the precise therapeutic [23].

In this review, the latest progress in polyphenol-based hydrogels in wound treatment and monitoring is comprehensively summarized. The construction strategies of polyphenol-based hydrogels are listed to help readers understand the construction principle of polyphenol-based hydrogels. The advanced functions of polyphenol-based hydrogels, including skin and tissue adhesion, antibacterial, anti-inflammatory, self-healing, photodynamic therapy, promoting wound healing, and wound monitoring, are briefly introduced. Then, we focus on the potential application of polyphenol-based hydrogels as sensor-based wound dressings, and the application prospect of machine learning in analyzing complex wound monitoring parameters for the accurate prediction of healing stage was also mentioned. Finally, the challenges of polyphenol-based hydrogel towards personalized wound management are pointed out. This review aims to summarize the latest developments in the field of polyphenol-based hydrogels and provide new insights for the design, fabrication, and application of the next-generation polyphenol-based hydrogel.

## 2. The Preparation and Classification of Polyphenol-Based Hydrogels

Polyphenols are one of the most widely distributed secondary metabolites found in plants containing at least one phenolic ring with one or more hydroxyl groups. So far, more than 8000 polyphenolic compounds have been extracted and identified [24]. According to their basic chemical structure characteristics, polyphenols are generally divided into five main categories: phenolic acids, flavonoids, lignans, stilbenes, and others [25]. Among them, flavonoids and phenolic acids account for over 90% of the total natural polyphenolic compounds [26]. Due to their antibacterial, antioxidant, anti-inflammatory, and anti-tumor properties, polyphenols have attracted great interest in the biomedical fields [27]. The multiple aromatic rings and hydroxyl group structures of polyphenol compounds allow them to react with various organic or inorganic substances [28]. However, the poor stability of polyphenolic compounds poses a significant challenge, as their structural integrity and physical properties are readily compromised by external factors—including temperature, pH, and light—leading to a consequent loss of bioactivity.

**Figure 1:**
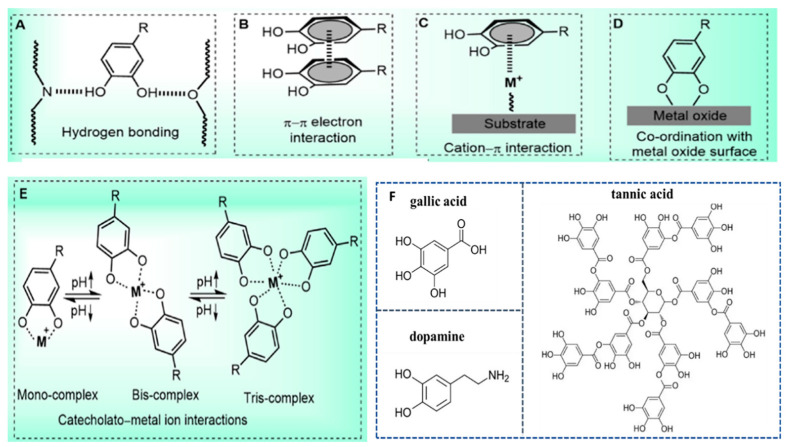
The crosslinking mechanisms of polyphenol-based hydrogels and molecular structure of typical polyphenol polyphenolic compounds: (**A**) hydrogen bonding interactions through catecholic –OH groups; (**B**) π–π electron interaction with another benzene ring; (**C**) cation–π interaction with positively charged ions; (**D**) coordination interactions with metal oxide surfaces; (**E**) formation of catecholate-metal ion complexes; (**F**) the molecular structure of tannic acid, dopamine, and gallic acid. Reproduced from reference [29] with permission from Elsevier, copyright 2018.

Polyphenol-based hydrogels are beneficial to improve the stability and bioavailability of polyphenolic compounds. In the polyphenol-based hydrogel systems, the three-dimensional structures are formed through physical or chemical crosslinking by multiple interactions of polyphenolic compounds, which endow hydrogels with a wide range of biomedical applications. As shown in Figure 1, the representative crosslinking mechanisms of polyphenol-based hydrogel include polymerization, π–π stacking interaction, cation–π bonding, hydrogen bonding, and electrostatic interactions, and the coordination of polyphenols with metal ions [29,30]. According to the crosslinking mechanisms, polyphenol-based hydrogels can be divided into physically crosslinked and chemically crosslinked hydrogels. Physical crosslinking primarily includes hydrogen bonding and metal coordination. The abundant phenolic hydroxyl groups in polyphenols can form extensive hydrogen bonds with donor/acceptor groups (e.g., carboxyl, amino, or ether groups) on other polymer chains, leading to the formation of hydrogels that often exhibit pH responsiveness. Furthermore, polyphenolic substances, especially those containing catechol groups, such as tannic acid, can form strong coordination bonds with metal ions (such as Fe^3^⁺, Cu^2^⁺, and Ca^2^⁺), thereby forming a metal-polyphenolic network. This crosslinking method is rapid and efficient, and can endow hydrogels with properties such as conductivity and self-repairing capabilities. Chemical crosslinking includes enzyme-mediated crosslinking, free radical polymerization, and click chemistry. These methods typically involve covalent bond formation between phenolic hydroxyl groups and other crosslinking components, resulting in stable polymeric networks.

In recent years, various polyphenols, including polydopamine (PDA) [31,32], tannic acid (TA) [33], gallic acid (GA) [34], epigallocatechin gallate (EGCG) [35], curcumin [36], and anthocyanin [37], have been utilized in hydrogel formation. Among these polyphenols, only a small number can form hydrogels independently under specific conditions, while the majority primarily act as crosslinkers or functional regulators to form composite hydrogels with other components. These components include natural polymers (such as gelatin, chitosan, hyaluronic acid, and alginate) and synthetic polymers (e.g., polyacrylic acid, polyethylene glycol, and polyvinyl alcohol). Therefore, according to the material source and composition, polyphenol-based hydrogels can be further categorized into three types: (1) Pure polyphenol self-assembled hydrogel: A small amount of polyphenols (such as certain tannic acid derivatives) can form a hydrogel through self-assembly under specific conditions, but such systems are relatively rare. (2) Polyphenol/natural polymer composite hydrogels: These hydrogels are formed by combining polyphenols with natural polymers. In these systems, polyphenols act not only as crosslinking agents but also as functional regulators, thereby enhancing antioxidant, antibacterial, and adhesion properties. This is the most extensively studied category. (3) Polyphenol/synthetic polymer composite hydrogels: Polyphenols are incorporated into synthetic polymer systems, so as to endow the originally inactive materials with biological activity.

To present the topic more clearly and intuitively, this section will classify polyphenol-based hydrogels based on the types of polyphenols used. The TA-based hydrogels, PDA-based hydrogels, and GA-based hydrogels are introduced in detail, and the molecular structures of tannic acid, dopamine, and gallic acid are displayed in Figure 1.

### 2.1. Tannic (TA)-Based Hydrogels

**Figure 2:**
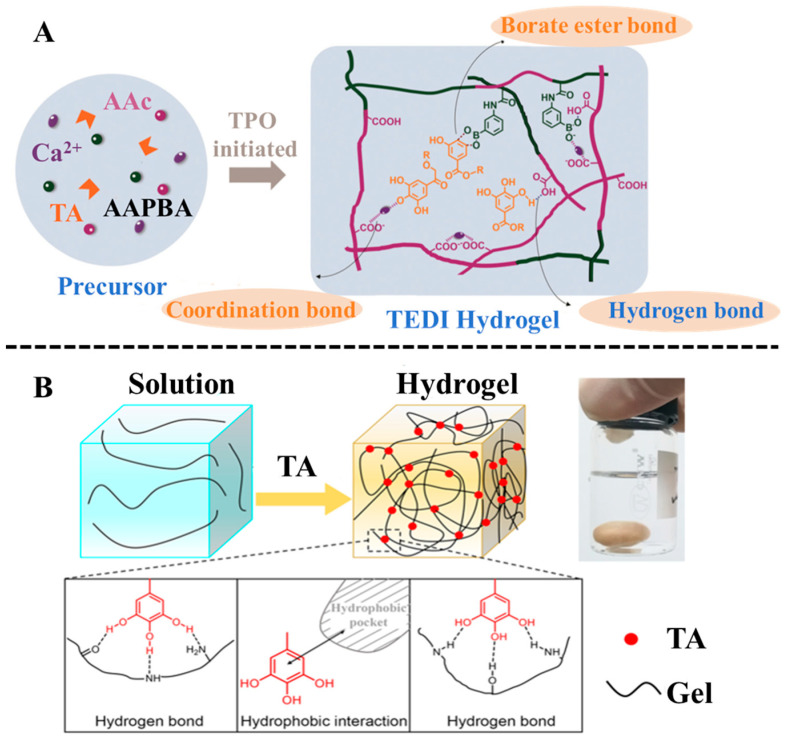
(**A**) Preparation process and chemical architecture of the tannic-acid-enabled dynamic interaction hydrogel. Reproduced from reference [41] with permission from the Royal Society of Chemistry, copyright 2021. (**B**) Formation process and mechanism of gelatin-TA hydrogel for fabrication of a self-powered strain sensor. Reproduced from reference [42] with permission from the American Chemical Society, copyright 2020.

Tannic acid is a polyphenolic compound derived from plants, and it is the most popular molecule among polyphenol compounds [38]. TA molecule with ten polyphenol groups owns a large molecular weight, leading to direct participation in the crosslinking of hydrogels by hydrogen bond interaction without further polymerization [39]. Yuan et al. [40] proposed a stepwise immersion method for the construction of gelatin-TA hydrogel depending on the hydrogen bonding between gelatin and TA, and the triple-helix structure of gelatin to form a physically crosslinked network. The proposed construction strategy avoided using chemical crosslinking agents. Upon deprotonation, TA forms oxygen centers with high electron density, enabling it to chelate metal cations. Utilizing this property, Mo et al. [41] developed a facile strategy to engineer an ultra-stretchable, highly adhesive, and self-healable hydrogel from TA, acrylic acid, and (3-acrylamidophenyl) boronic acid (Figure 2A). The conventional covalent crosslinking was replaced by the multiple dynamic interactions of TA, including coordination bonds between TA and calcium ion, hydrogen bonds formed in TA and acrylic acid, and borate ester bonds between TA and (3-acrylamidophenyl) boronic acid. Their prepared hydrogel could serve as a wearable, flexible strain sensor to accurately detect the motion of the human body. Owing to their multifunctional properties, TA-based hydrogels are promising candidate materials for portable and wearable electronic devices. A notable example is the gelatin-TA hydrogel developed by Wang et al. [42] via a simple one-step method to fabricate a self-powered strain sensor (Figure 2B). This hydrogel leverages hydrophobic interactions and hydrogen bonding between TA and gelatin, resulting in excellent elongation, rapid autonomous self-healing, and high healing efficiency. As a result, the sensor exhibits excellent responsiveness and flexibility. In summary, TA is widely used to construct various functional hydrogels due to its outstanding biological and physicochemical properties.

### 2.2. Polydopamine (PDA)-Based Hydrogels

**Figure 3:**
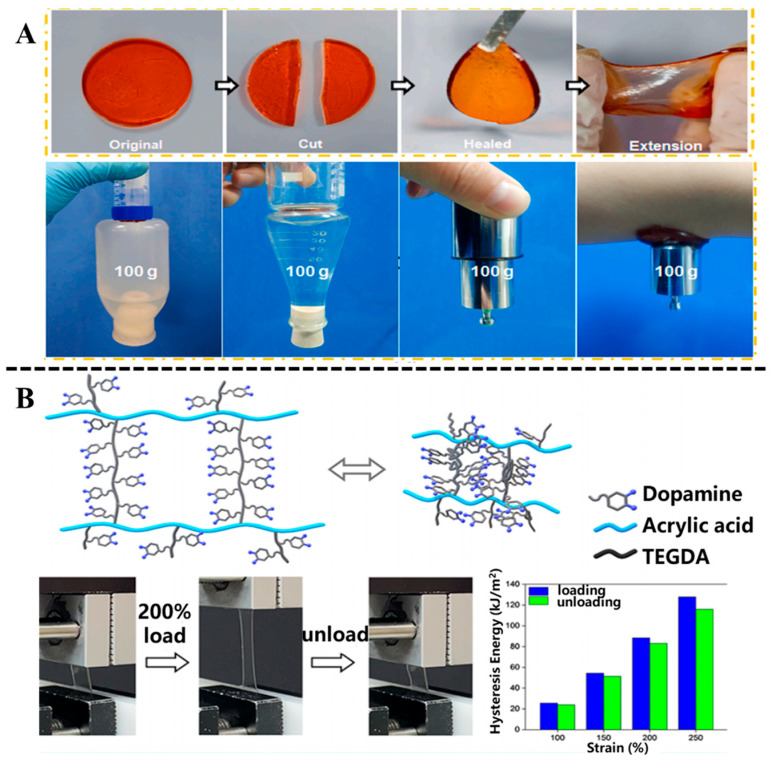
(**A**) Self-healing properties of the PDA-PAM/Mg^2+^ hydrogel and the macroscopic adhesion of the hydrogel to various substrates. Reproduced from reference [50] with permission from Elsevier, copyright 2022. (**B**) Schematic illustration of network structure of the dual-crosslinked hydrogel and the cyclic tensile test and hysteresis energy of the hydrogel. Reproduced from reference [52] with permission from the American Chemical Society, copyright 2022.

Polydopamine (PDA), as a key catecholamine neurotransmitter, contains a catechol group with catechol structure and a primary amino group. Its catechol groups exhibit strong adhesion, redox activity, and good biocompatibility [43]. DA or PDA is widely used to construct hydrogels, which are introduced into 3D network structures as crosslinking agents or functional components [44,45]. Dopamine forms a hydrogel network with various polymers such as gelatin [46], chitosan [47], polyethylene glycol [48], or metal ions [49] through various interactions such as hydrogen bonding, π–π stacking, and Schiff base reaction. For example, Guo et al. [50] developed a kind of composite hydrogel composed of polyacrylamide (PAM), PDA, and Mg^2+^ with favorable self-healing and adhesive capability by the interactions between the amino groups of PMA and PDA, and catechol groups in PDA and the amino groups in PAM (Figure 3A). Schiff-base reactions can be applied to the crosslinking of polyphenol-based hydrogel systems, which increases the strength of hydrogels. Li et al. [51] designed a dual-network-structure-engineered hydrogel by using hyaluronic acid (HA), polyethylene glycol diamine (PEG), and PDA. HA-grafted PEG was used to prepare crosslinked HA-PEG hydrogel as the first crosslinked network; subsequently, PDA and HA were reacted with Schiff base to prepare highly crosslinked PDA-CHO-HA hydrogel as the second crosslinked network. Their prepared hydrogel demonstrated mechanical properties similar to the nucleus pulposus and displayed potential application in the treatment of intervertebral disk degeneration. Dopamine can participate in the preparation of double-crosslinked network hydrogels through the synergistic effect of covalent and non-covalent crosslinking. Han et al. [52] developed a dual-crosslinked hydrogel employing a dopamine-modified, acrylate-terminated crosslinker, termed tri(ethylene glycol) diacrylate-dopamine (Figure 3B). The resulting hydrogel exhibited robust tissue adhesion under moist conditions and high mechanical resilience. In conclusion, incorporating polydopamine (PDA) into polymeric networks provides an effective strategy to enhance the biological performance of hydrogels, showing considerable promise for a wide range of biomedical applications.

### 2.3. Gallic Acid (GA)-Based Hydrogels

**Figure 4:**
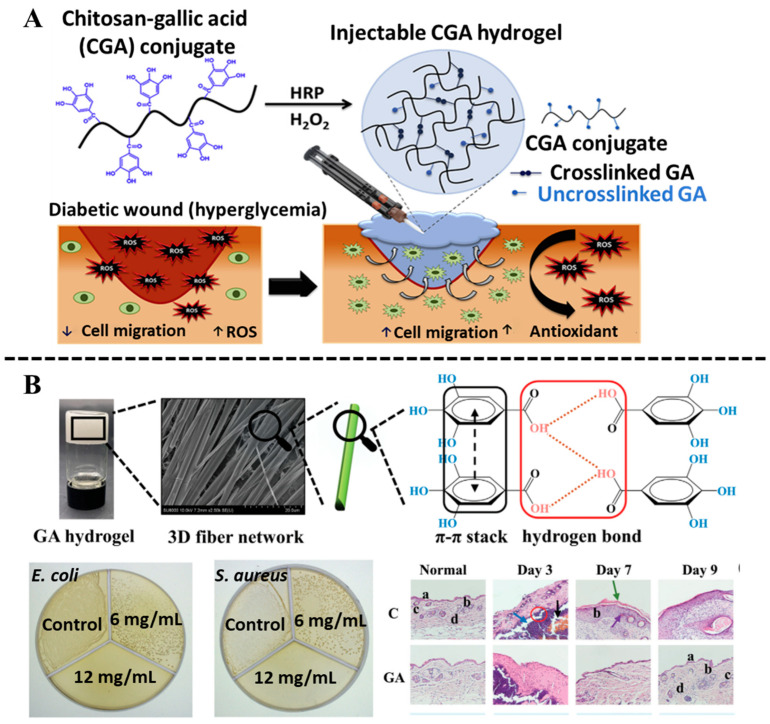
(**A**) Preparation of CGA hydrogel through H_2_O_2_-mediated crosslinking. Reproduced from reference [57] with permission from Elsevier, copyright 2020. (**B**) Self-assembly of the GA hydrogels, and antibacterial activity of GA hydrogels against *E. coli* and *S. aureus* treated with different concentrations, as well as the in vivo evaluation of GA hydrogel for wound healing. Reproduced from reference [58] with permission from Wiley, copyright 2022.

Gallic acid (GA), as a plant-based polyphenol, can be found in tea, peony, Chinese nutgall, and other plants [53]. It is proven that GA owns numerous pharmacological activities, including antioxidant, antibacterial, anti-inflammatory, and metal ion-chelating ability [54]. However, the characteristics of low bioavailability, poor lipophilicity, and easy oxidation restrict its further applications in clinics. Many efforts have been made to improve the biological utilization of GA, such as preparing nanoparticles or constructing hydrogels [55,56]. In the fabrication of hydrogels, GA can function either as a crosslinking agent or a bioactive additive. Tran et al. [57] demonstrated that GA could be grafted onto the chitosan backbone via EDC/NHS chemistry, resulting in an in situ crosslinkable GA–chitosan hydrogel with ROS-scavenging capability. The mechanical properties and microstructure of this hydrogel could be tuned by adjusting the H_2_O_2_ concentration (Figure 4A). The three hydroxyl groups in GA play a critical role in hydrogel formation through intermolecular hydrogen bonding and coordination interactions. Huang et al. [58] developed an efficient method to construct a self-assembled GA hydrogel based on *π*-*π* stacking and hydrogen bonding, revealing that both the three hydroxyl groups and one carbonyl group in the GA molecule are essential for hydrogel formation (Figure 4B). Their constructed GA hydrogel showed anti-inflammatory, antibacterial effects, and the ability to promote wound healing without cytotoxicity. Likewise, Zhou et al. [59] designed a kind of multifunctional hydrogel with favorable injectability, electrical conductivity, rapid shape adaptation, and antimicrobial activity through Schiff base reaction between gelatin quaternary ammonium salt, GA, and sodium dialdehyde alginate. In addition, CuS@TA-Fe nanoparticles were embedded with the hydrogel, which enables the hydrogel to exhibit anti-inflammatory and antioxidant capabilities via photothermal (PTT) and photodynamic (PDT) effects. GA-based hydrogels, with their unique advantages, show great potential in the field of biomedicine and provide efficient solutions in the biomedical field, such as precision treatment, infection control, and wound healing.

### 2.4. Other Polyphenol-Based Hydrogels

In addition to the aforementioned polyphenol-based hydrogels, various natural polyphenols (such as lignin, anthocyanin, tea polyphenols, ellagic acid, quercetin, luteolin, etc.) have also been used in the preparation of functional hydrogels. Among these, lignin stands out as the second most abundant natural polymer on Earth after cellulose. It is widely found in the cell walls of higher plants, where it collaborates with cellulose and hemicellulose to form the fundamental structural matrix of plant tissues. Natural lignin possesses a polyphenol-like architecture with numerous aromatic rings, phenolic hydroxyl, and methoxyl groups. However, as each aromatic unit generally contains only one phenolic hydroxyl group, natural lignin exhibits limited polyphenol-like properties. To enhance its polyphenolic functionality, Sun et al. developed an efficient and eco-friendly demethylation strategy using a low-cost bifunctional protic ionic liquid under mild and halogen-free conditions, facilitating the conversion of lignin into value-added polyphenols [60]. By utilizing this modified material, Du et al. engineered a multifunctional hydrogel (DLLMH) based on liquid metal (LM) nanospheres encapsulated by demethylated lignin (DL). The resulting DLLMH exhibits integrated superior mechanical strength, high electrical conductivity, strong adhesion, and rapid self-healing behavior [61]. Furthermore, Li et al. fabricated a dual-network hydrogel composed of lignin/poly (N, N-dimethylacrylamide) and sodium alginate/Ca^2^⁺, which demonstrated remarkable rupture resistance, impact tolerance, and high conductivity [62]. Owing to its ultra-tough and highly stiff characteristics, the lignin-based hydrogel shows significant application potential in high-demand environments such as extreme sports and advanced wearable technology.

Tea polyphenols (TPs) with polyhydroxy structure exhibit notable biological activities, including antioxidant, antibacterial, antiviral, and antitumor effects. These properties, combined with their ability to participate in diverse assembly mechanisms such as hydrogen bonding, metal coordination, and π-π stacking, make them promising building blocks for the synthesis and formation of functional hydrogels. For example, Dong et al. developed a double-network hydrogel through hydrogen bonding between tea polyphenol/glycerol and a photo-crosslinked network composed of N-acryloyl glycinamide, gelatin methacrylate, and nanoclay [63]. The prepared hydrogel exhibited inherent anti-ultraviolet, antioxidant, antibacterial activity and excellent water retention, and accelerated wound healing by promoting wound closure, collagen deposition, angiogenesis, and tissue remodeling.

Regarding other small-molecule polyphenols such as ellagic acid, quercetin, and luteolin, their low molecular weight prevents them from independently forming hydrogels, unlike high-molecular-weight polymers. Nevertheless, due to their small size and high bioactivity, these compounds can be ingeniously incorporated into polymer networks to act as both crosslinking promoters and functional regulators. This integration facilitates the development of a new generation of multifunctional hydrogels with improved mechanical strength, smart responsiveness, and enhanced biological activities. This approach represents a highly significant research direction in the field of biomaterials.

In summary, polyphenols can be combined with natural polymers and also are crosslinked with synthetic polymers to construct multifunctional hydrogels through physical or chemical methods. Polyphenol-based hydrogels improve the bioavailability of polyphenolic compounds and greatly broaden the application fields of polyphenolic compounds. The research on polyphenol-based hydrogels needs the deep involvement of multiple disciplines. Through sophisticated molecular design, multiple functional integration, and strict clinical evaluation, polyphenol-based hydrogels are expected to move from laboratory to practical applications.

## 3. The Applications of Polyphenol-Based Hydrogels

At present, a wide variety of hydrogels, such as polyvinyl alcohol (PVA), polyacrylic acid (PAA), and alginate gels, are extensively used in biomedical applications, including drug delivery, wound dressings, tissue engineering, contact lenses, fillers, etc. However, most of the hydrogels mainly serve as physical scaffolds, barriers, or water storage devices, and they lack the ability to actively modulate the wound microenvironment by suppressing inflammation, scavenging free radicals, or promoting cellular processes essential for healing. In contrast, polyphenol-based hydrogels represent a significant advance toward intelligent and interactive wound care platforms. Taking advantage of their inherent biocompatibility, potent antioxidant and antibacterial properties, tunable adhesion, and stimulus-responsive behavior, polyphenol-based hydrogels not only provide essential physical supports but also actively promote wound healing. Moreover, they serve as versatile matrices for constructing responsive drug delivery systems that enable controlled release of therapeutic agents in response to wounds [64]. Except for treatment, polyphenol-based hydrogels also play a crucial role in real-time wound monitoring, which is beneficial for precise management and prevention of wound infections. It is noteworthy that the polyphenol-based hydrogels can be used as the interface material of a biosensor to improve the detection performance [65]. By loading color probes such as pH indicators into the hydrogel network, the hydrogel can produce visible color changes with the changing of the wound microenvironment [66,67]. This visual sensing without complex equipment can provide immediate parameters for timely intervention and treatment of wounds, and the efficiency of wound management is significantly improved. On the other hand, the three-dimensional network structure of polyphenol-based hydrogels allows the electrochemically active probes to be efficiently immobilized, and the strong adhesion of polyphenol-based hydrogels to the irregular wound surface ensures the achievement of a stable electrochemical signal. The changing of biomarkers in the wound microenvironment can be converted into the changing of electrical signals, such as current, impedance, or potential, which provide real-time data for precise diagnosis and treatment of wounds. In this section, the applications of polyphenol-based hydrogels in wound monitoring and treatment are systematically summarized.

### 3.1. Applications of Polyphenol-Based Hydrogels in Wound Healing

Skin, as the largest organ of the human body, is a key barrier to resist pathogen invasion, prevent water loss, regulate body temperature, and so on [68]. Once skin injury occurs, the skin will lose its most important protective function and be susceptible to infection by pathogenic bacteria [69]. Even worse, drug-resistant bacterial infections hinder the healing process and greatly increase the risk of chronic and even fatal systemic infections, posing a severe threat to wound therapy [70]. Polyphenol-based hydrogels, as a new wound dressing, have unparalleled advantages in wound healing due to broad-spectrum antibacterial, excellent antioxidant, and anti-inflammatory properties from polyphenolic compounds, as well as continuous drug release ability from the hydrogel matrix. More and more studies have been reported about the applications of polyphenol-based hydrogels in the treatment of bacteria-infected wounds.

**Figure 5:**
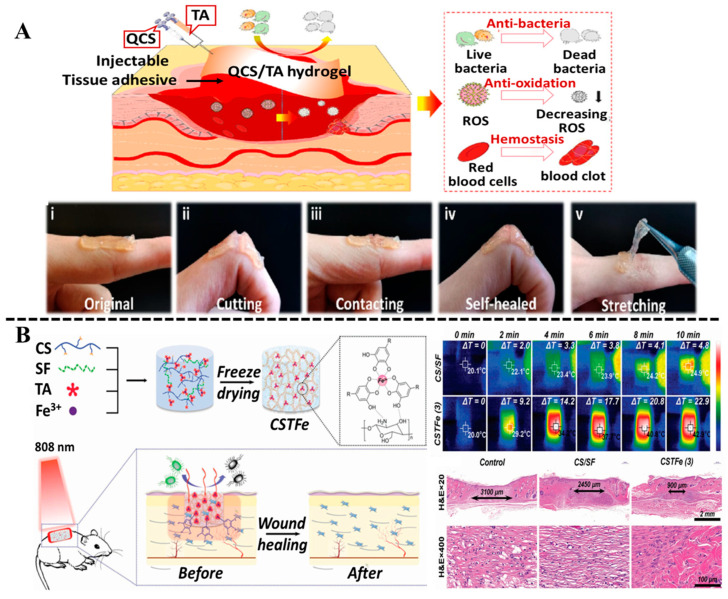
(**A**) QCS/TA hydrogels with excellent injectable, self-healing, and adhesive properties for wound healing. Reproduced from reference [71] with permission from the American Chemical Society, copyright 2022. (**B**) The preparation of multifunctional and recyclable photothermally responsive cryogels and their application as wound dressing materials. Reproduced from reference [73] with permission from Wiley, copyright 2019.

Considering the structure of hydrogel is inevitably deformed by body movement or external force, the self-healing and tissue-adhesive hydrogels capable of preventing detachment from skin are more conducive to long-term wound treatment. Due to the rich phenolic hydroxyl structure, polyphenols play a crosslinking role in hydrogels through various covalent and non-covalent interactions, which endow hydrogels with high adhesion and self-healing properties. A multifunctional quaternary ammonium chitosan (QCS)/tannic acid (TA) hydrogel with excellent injectable, self-healing, and adhesive properties was fabricated through a facile and one-step approach by Yao and his colleagues [71]. The experimental results showed that the QCS/TA hydrogel exhibited excellent biocompatibility, strong antibacterial activity, and scavenging activity of reactive oxygen species, which was expected to be used as a first-aid dressing material for rapid hemostasis and skin wound repair (Figure 5A). In addition, You et al. developed a polyphenol-enhanced wet adhesion hydrogel with the synergistic effect of mechanical activation and ROS scavenging, which was constructed through the copolymerization of acrylic acid (AAc), 1-vinylimidazole (VI), and tannic acid (TA), for promoting the healing of infected diabetic wounds [72]. Peel and lap-shear tests suggested that the adhesion energy increased with the rise in TA content, and the addition of TA enhanced the toughness and wet tissue adhesion of the hydrogel.

In the past decades, owing to the overuse of traditional antibiotics, bacterial-drug resistance in wound infections has become severe, making wound infection difficult to control, even delaying healing process of infected wounds [74]. By utilizing the local heat produced by photothermal conversion materials under near-infrared light irradiation, phototherapy, including photodynamic therapy (PDT) and photothermal therapy (PTT), is an effective antibiotic alternative therapy to fight against bacterial infections [75]. Hydrogel combined with phototherapy has gained great attention in wound treatment owing to its low irritation, high efficiency, and good antibacterial performance [76,77]. Although some photothermal materials like noble metal nanoparticles [78], organic small molecules [79], carbon-based nanomaterials [80] have already been introduced into hydrogel for antibacterial therapy. However, some shortcomings, including low photothermal efficiency, poor biocompatibility, complexity, and high cost in fabrication, still remain to be overcome. Recent studies have shown that natural polyphenols can chelate with metal ions to form complexes with strong near-infrared light absorption [81]. The Fe^3+^, as an essential element for the human body, could serve as multivalent sites to chelate with TA to form stable complexes [82]. Some studies indicated that the TA/Fe^3+^ complexes had good photothermal efficiency due to the broad near-infrared absorption, and could be loaded on hydrogel networks to fabricate antibacterial dressings for wound healing [83,84]. For example, He et al. [85] constructed an ultra-stretchable, self-healable, and skin-adhesive hybrid hydrogel wound dressing by simple copolymerization of acrylamide, 3-acrylamido phenylboronic acid, chitosan, and TA/Fe^3+^ complex (TFe). Benefiting from the high photothermal conversion efficiency of the TFe, the hydrogel exhibited excellent bactericidal activity against S. aureus and E. coli, and effectively eliminated bacterial biofilms in the wound bed to accelerate the healing process of infected wounds. A multifunctional cryogel with hemostasis, exudate absorbance, antibacterial effects, promotion of cell proliferation, and recycled utilization was proposed by Zhang et al. [73]. The cryogel was obtained by the one-step mixing of natural products chitosan, silk fibroin, TA, and Fe^3+^ via the freeze-drying method. Because of the introduction of the TA/Fe3+ complex, the cryogel showed excellent photothermal sterilization ability and displayed excellent antibacterial activity to both Gram-negative and Gram-positive bacteria, owing to the high photothermal transition activity (Figure 5B). In addition, the in vivo trauma model experiment proved that the cryogel accelerated wound repair due to the promotion of cell adherence and proliferation by the TA/Fe^3+^ complex.

To sum up, polyphenol-based hydrogels show powerful advantages in the construction of skin or tissue adhesive and photothermal antibacterial wound dressings. However, polyphenol-based hydrogels for wound healing are still far from clinical practice. The main challenges include the following: (1) polyphenols are easily oxidized, affecting the long-term stability and performance; (2) it is difficult to control temperature precisely in photothermal therapy to avoid thermal damage to healthy tissues; (3) there is a lack of systematic evaluation of long-term in vivo biosafety. Overcoming these challenges is conducive to promote polyphenol-based hydrogel to the clinical transformation of wound treatment.

### 3.2. Polyphenol-Based Hydrogels for Colorimetric Sensing of Wound

In recent years, colorimetric detection technology, as a visual detection way for wound monitoring, has developed rapidly since it can reflect the physiological state of wounds based on the color response without requiring additional large equipment [86,87,88]. Colorimetric sensing has the advantages of easy operation, rapid response, and low cost. The basic principle of colorimetric sensing for wound monitoring is based on the reversible or irreversible color changes in colorimetric materials induced by specific biomarkers in the wound microenvironment, such as pH, enzymes, reactive oxygen, or bacterial metabolites. The color changes can be captured by the naked eye or a digital camera; subsequently, the information from the collected images is processed by machine learning, such as convolutional neural networks (CNNs). The three-dimensional network structure of the hydrogel allows the efficient loading of the color probes, which are beneficial for the integration of pH sensing. More importantly, the color change can be observed without removing the hydrogel, thereby avoiding the secondary damage to the wound like the traditional wound dressings.

It is well documented that the pH of normal skin ranges from 5.5 to 6.0, while the pH of an infected wound increases owing to the alkaline substances secreted by bacteria or fungi [89]. Therefore, the pH monitoring of the wound bed is deemed one of the most remarkable biomarkers as an important indicator of the status of the wound throughout the healing process. It also provides a forewarning of bacterial infection. Various types of colorimetric dyes and fluorescent probes have been discovered with pH-sensing functions [90,91]. Synthetic pH indicators, including bromothymol blue, phenol red, and bromocresol purple, have the advantages of sensitive color response and good stability towards light, chemicals, or thermal environment [92]. However, synthetic indicators may have biological toxicity and poor biodegradability, which limit their application in wound pH monitoring [93]. Plant-based pH indicators derived from roots, leaves, and flowers of various plants exhibit different colors with the change in environmental pH. These natural pH probes have the advantages of being non-toxic, highly biocompatible, and environmentally friendly, avoiding the safety concerns of synthesized indicators [94,95]. Among plant-based pH indicators, polyphenolic compounds have become one of the most important candidates owing to their pH-dependent electronic transition characteristics. In addition, polyphenolic compounds can be embedded into the hydrogel network to achieve a dual function of pH monitoring and antibacterial activity, which assists wound healing.

**Figure 6:**
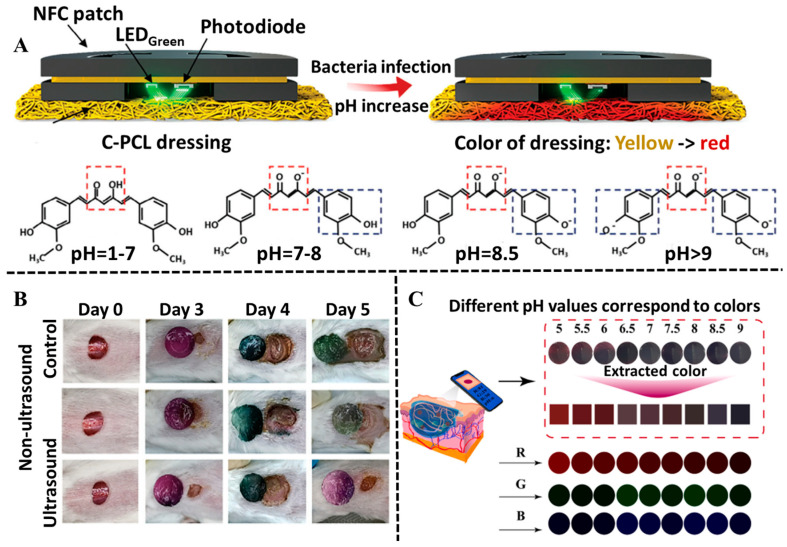
Polyphenol-based hydrogels in the application of wound colorimetric sensing. (**A**) Working mechanism of the wireless, battery-free, optoelectronic diagnostic sensor integrated with a pH-colorimetric wound dressing and the alterations in curcumin chemical structure according to pH levels. Reproduced from reference [99] with permission from Wiley, copyright 2024. (**B**) The color changes in anthocyanin/cefazolin sodium (CS)-loaded poly (L-lactic acid) microcapsules hydrogels at different time points in the infected wound models on mice. Reproduced from reference [101] with permission from Wiley, copyright 2024. (**C**) The pH of the multifunctional MOFs hydrogels responds to color change and the related trichromatic (RGB) colors. Reproduced from reference [102] with permission from Elsevier, copyright 2024.

Curcumin, as an important polyphenolic compound isolated from turmeric, not only has pH-indicator ability, but also contributes to accelerating the healing process [96,97]. Curcumin in an acidic environment is mainly in the keto form, while it gradually deprotonates to form the enol form in an alkaline environment. With the pH changing from 6~8, a remarkable color change in curcumin is observed from yellow to red-brown. To date, curcumin has been used as a good pH indicator to identify wound healing by the naked eye [98]. For example, Cho et al. designed a wireless, battery-free, optoelectronic diagnostic sensor integrated with a pH-colorimetric wound dressing composed of curcumin and polycaprolactone-based composite material [99]. In addition, the integrated optoelectronic-based diagnostic sensor enabled the color change in curcumin to be quantitatively monitored in a real-time fashion; thereby, the progress of wound healing was tracked by visual discrimination without needing in-depth medical knowledge (Figure 6A). In another study, curcuma longa extract (CLE) was loaded in hydroxyethyl cellulose hydrogel to fabricate a smart pH-sensitive wound dressing. The hydrogel could exhibit a visual color change from yellow to reddish-brown at acidic and basic media, so as to indicate the pH of the wound bed with an easily stripped-off property [100].

Anthocyanins extracted from fruits or vegetables such as purple sweet potato, purple cabbage, blueberry, etc., are water-soluble pigments, which demonstrate excellent pH response sensitivity [103]. The color-changing range of anthocyanins is between pH 2 and 12, varying with the source of anthocyanins. Studies have confirmed that anthocyanins from purple cabbage possess superior stability compared to those from other sources, distinguished by a wider range of pH-responsive colors: pink at pH 3, violet at pH 5, blue at pH 7, and green at elevated pH [104]. There are a great number of investigations about the integration of anthocyanins into hydrogels for pH sensing [105,106,107]. The loaded anthocyanin hydrogels could change color according to the pH value of the wound exudate. Thus, wound pH can be determined by observing color with the naked eye, so as to achieve real-time, in situ, and visual monitoring. As a representative example, Huang et al. created a multifunctional hydrogel dressing featuring anthocyanin for colorimetric pH sensing and poly (L-lactic acid) microcapsules for controlled drug release under ultrasound [101]. This setup not only enables visual wound assessment through color changes but also provides on-demand therapeutic action. The pH response, detectable across a range of 5–9, is precisely measured by analyzing RGB values from smartphone images of the dressing (Figure 6B). Thereby, wound infection was effectively detected by monitoring pH levels. In another study, Zhao et al. designed a multifunctional MOF hydrogel containing blueberry anthocyanin to monitor wound healing through color changes in response to environmental pH with a range of 5–9 [102] (Figure 6C). In addition, an innovative “Tri-Act” bilayer hydrogel with functions of visual monitoring of wound infection, immune regulation, and angiogenesis promotion was developed by Zhang et al. [108]. The upper layer of the hydrogel utilized the pH-responsive color-changing properties of anthocyanin-rich extracts and was able to visually monitor wound infection, which simplified the process of diagnosing wound infections.

In short, the key advantage of polyphenol-based hydrogels for pH monitoring is that the polyphenol itself possesses pH response characteristics, which can cause visual color changes through protonation/deprotonation. Thus, it realizes real-time and visual monitoring of wounds. In the future, the development direction of polyphenol-based hydrogels for wound colorimetric sensing should focus on the following areas: one is utilizing the inherent biological activities of polyphenols, such as adhesion and antibacterial activity, to achieve the dual function of wound sensing and wound healing and another is using smart phone image analysis technology to improve the sensitivity of color signal, so as to promote the development of portable wound care equipment.

### 3.3. Polyphenol-Based Hydrogels for Wound Electrochemical Sensing

Biological markers, such as uric acid, lactate, inflammatory factor, infected bacteria, and physical parameters such as temperature, pH, and oxygen, can be regarded as valuable indicators of the biological state of the wound [109,110]. Effective skin treatment and continuous wound monitoring have always been a major challenge in biomedical issues. Currently, wearable electronic devices with the ability to monitor various physiological signals have attracted much attention in the field of biomedicine. The key issue of wearable electronic devices for wound sensing is to achieve adhesion between sensors and skin due to the incompatibility between electronics and biology. With excellent biocompatibility, controllable conductivity, high tensile strength, and self-healing ability, conductive hydrogels have become an ideal material for building wearable electronic devices [111].

**Figure 7:**
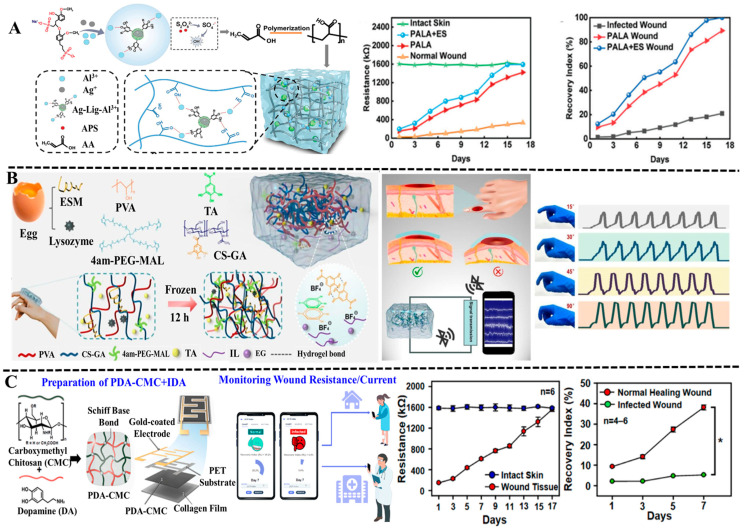
Polyphenol-based hydrogels in applications of wound electrochemical sensing. (**A**) The bimetallic phenolic network hydrogel has self-adhesive, rigid, antibacterial, and conductive abilities, and the resistance values of intact skin and infected wound were obtained at different times during the healing process. Reproduced from reference [113] with permission from Wiley, copyright 2025. (**B**) The multifunctional organo-hydrogel flexible sensor for monitoring of wounds at finger joints. Reproduced from reference [114] with permission from the American Chemical Society, copyright 2024. (**C**). The working mechanism of smart electroactive dressing, the resistance changes in wound tissue, and the differences in wound recovery indices between a normally healing wound and an infected wound. Reproduced from reference [115] with permission from Elsevier, copyright 2022.

Polyphenol-based hydrogels with strong adhesion, self-healing ability, and biocompatibility can be combined with flexible conductive materials such as graphene, conductive polymers, and metal nanoparticles to build multi-functional conductive hydrogels and intelligent sensor systems [65,112]. The monitoring of joint wounds presents a greater challenge since it requires wearable electronic devices to have excellent mechanical properties, self-healing ability, and long-term adhesion due to frequent activities. In recent years, polyphenol-based conductive hydrogels have been reported to possess the capability for strain-sensing and could be applied in sports-related wounds. They can record real-time signals such as the strain and resistance of the wound, achieving synchronous monitoring of the wound healing status and local muscle movements. For example, Yang et al. prepared a novel bimetallic phenolic network hydrogel with high antimicrobial, electrical conductivity, and bio-tissue adhesion [113]. The designed hydrogel sensor could sense the movement of different body parts. Based on sensitive and stable sensing properties of the hydrogel, they also developed an antimicrobial bioelectronic device with the ability of electrotherapeutic synergy and wound monitoring. The experiment results indicated that the resistance decreased when the skin was damaged, and resistance increased gradually during healing. On the basis of the resistance change, the wound healing process could be monitored in real-time (Figure 7A).

Machine learning algorithms have played an important role in the intelligent analysis of wound monitoring due to their powerful ability of pattern recognition, the processing of nonlinear data, and prediction capability of adaptive learning [116,117]. The machine learning-enabled wound monitoring system transforms into an intelligent diagnosis tool, improving the efficiency, accuracy, and predictability of wound management. For example, Li et al. designed a multifunctional organo-hydrogel flexible sensor to monitor the real-time movement amplitude of wounds by using poly (vinyl alcohol), gallic acid-grafted chitosan, tannic acid, eggshell membrane, lysozyme, and tetra-armed poly (ethylene glycol) maleimide [114]. The organo-hydrogel sensor exhibited good antimicrobial property and biocompatibility, and could provide emergency cooling after fireworks burns and accelerate wound healing. Notably, a deep learning model based on a lightweight CNN architecture was developed to monitor finger joint injuries for wound healing monitoring by analyzing the resistance signals (Figure 7B).

Bacterial infections, especially drug-resistant bacterial infections, pose a serious threat to wound healing [118,119,120]. The invasion of bacteria into wounds will trigger inflammatory reactions, damage newly formed tissues, and ultimately delay the healing process. It may even invade deep tissues or blood, causing systemic infections. Nhien and colleagues developed an engineered electroactive dressing composed of PDA-crosslinked CMCS and an interlaced array electrode to accelerate wound regeneration and monitor the progress of healing [115]. The PDA containing a catechol group ensured the tight adhesion to a skin wound, and the IDA electrode detected the resistance of the wound tissue, achieving continuous monitoring of the healing process of the wound. As is well known, bacterial biofilms are electroactive, and their presence can lead to a decrease in resistance. Their experiment found that the wound recovery index (RI) showed a significant decline once the wound was infected by E. coli. By observing the warning signal on the smartphone via the Wi-Fi system, medical staff and patients could immediately discover and treat the bacterial infections of the wound (Figure 7C).

In summary, taking advantage of the adhesion, antibacterial properties of polyphenols, and sensing ability of conductive materials, conductive polyphenol-based hydrogels have great application potential in the monitoring of joint movement strain and wound motion, as well as the in situ sensing of bacteria-infected wounds. Conductive polyphenol-based hydrogels have the capability to fit dynamic wounds and provide real-time feedback on physiological status of the wound. However, there are many challenges for conductive polyphenol-based hydrogels in regard to the application in wound monitoring. The mechanical strength and conductivity may decline during the process of long-term wearing. In addition, detection accuracy and range are susceptible to being affected by complex wound environments. It is necessary to make breakthroughs in the stability of hydrogel, the design and fabrication of anti-biological pollution sensing interface, the integration technology of flexible electronics, and combination of the artificial intelligence technology to promote conductive polyphenol-based hydrogels for intelligent wound management.

## 4. Polyphenol-Based Hydrogel Microneedles for Wound Treatment and Monitoring

Microneedles (MNs), consisting of an array of microscale needles, can penetrate the skin without touching blood capillaries and nerve endings [121]. MNs have attracted widespread attention in wound treatment due to their non-invasiveness, painless, controllable drug delivery, the capability of breaking down bacterial biofilms, and so on [122,123,124]. MNs fabricated by using responsive materials can monitor wound conditions, so as to deliver drugs on demand according to the wound healing states. In this section, the fabrication method and commonly used material of MNs, and the application of polyphenol-based hydrogel MNs for wound treatment and monitoring, were discussed.

**Figure 8:**
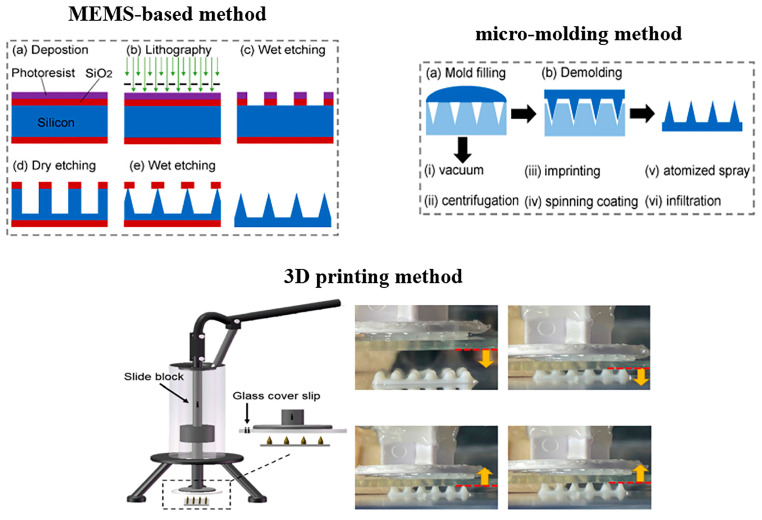
The construction methods of MNs including the MEMS-based method, micro-molding method, and 3D printing method. Reproduced from reference [125] with permission from Elsevier, copyright 2023, and reference [136] with permission from Elsevier, copyright 2020.

As shown in Figure 8, the construction methods of MNs mainly include MEMS-based methods, micro-molding methods, and 3D printing methods [125]. MEMS-based methods, with the advantage of high precision, are able to fabricate conical [126], pyramid-like [127], and hollow needles [128]. However, this preparation process is time-consuming and expensive, and is not able to produce the MNs with a complicated structure. Micro-molding, which has the advantages of being simple, inexpensive, and highly reproducible, is suitable for fabricating the MNs by using natural and synthetic polymers or hydrogels [129,130]. In addition, complicated structured MNs can be produced by this method. Owing to the surface tension force, the mold-filling is a key issue in the processing. To solve this problem, several methods, including vacuum [131], centrifugation [132], imprinting [133], and spinning coating [134], were developed by researchers. For 3D printing, photopolymerization is a widely used method to produce MNs, and the fused deposition modeling method is rarely adopted to construct MNs [135]. Through digital modeling, 3D printing is able to construct MNs with sophisticated structures and personalized shapes. At present, 3D printing technology makes it difficult to achieve large-scale MN production.

**Figure 9:**
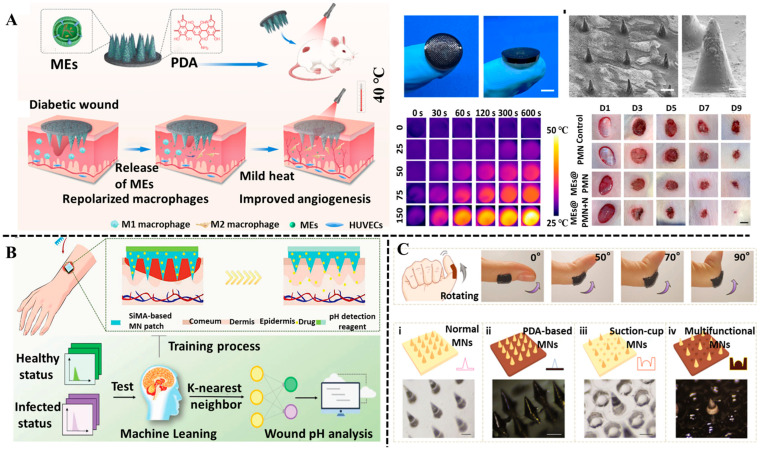
Polyphenol-based hydrogel MNs in the application of wound treatment and monitoring. (**A**) The double-layer microneedle-based wound dressing for treating diabetic wounds based on photothermal effect of PDA. Reproduced from reference [147] with permission from Elsevier, copyright 2023. (**B**) The pH-response and machine learning-assisted MNs sensing patch for wound treatment and visual monitoring. Reproduced from reference [148] with permission from Wiley, copyright 2024. (**C**) The morphology and adhesive characterization of bioinspired MNs on the knuckle. Reproduced from reference [146] with permission from Amer Assoc Advancement Science, copyright 2020.

According to application requirements, MNs can be made by using various materials such as metals [137], silicon [138], ceramics [139], and polymers [140]. MNs made of metal, silicon, or ceramics have excellent mechanical strength and penetration ability, making them suitable for drug delivery or as long-term implanted electrodes. However, they are not easily degraded under conventional conditions, and have relatively complex designs and expensive manufacturing costs. Compared with metal, polymer-based MNs demonstrated great application potential in wound healing owing to their excellent biocompatibility, degradability, and diverse drug loading capabilities [141]. Among polymer-based MNs, hydrogel-based MNs made by hyaluronic acid, chitosan, or hyaluronic acid methacrylate have shorter degradation durations and relatively low mechanical properties, beneficial for the rapid release of drugs [142]. In addition, by adjusting the density of the polymer network, hydrogel-based MNs with controlled drug release can be designed according to the molecular size of the drug. However, most hydrogel-based MNs are easy to fall off from the skin, and the poor adhesion ability restricts practical applications in wound treatment, especially for the joint parts with a large range of motions. Several studies reported that polyphenol-based hydrogel could serve as a practical material to fabricate MNs with good adhesion to skin [143,144,145]. For example, inspired by adhesion mechanisms of mussel byssi and octopus tentacles, a kind of bioinspired MNs with ideal adhesive and antibacterial ability was proposed by Zhang et al. [146]. In their designed MNs, polydopamine (PDA) hydrogel acted as the flexible base of the MNs, endowing the MNs with strong adhesion to the skin. In addition, the loaded polymyxin, both in the hydrogel tip and the PDA base, made the MNs possess bacteria-resistant ability. The experiment results indicated that the MNs exhibited excellent adhesion to knuckles and good antibacterial activity against E. coli (Figure 9A). It is proven as a promising candidate in wearable biomedical systems or wound treatment.

Nowadays, hydrogel-based MNs have emerged as promising tools for the treatment of wound infection [149]. Polyphenols, as bioactive molecules, can be incorporated in hydrogel-based MNs to fabricate multifunctional MNs with effective antibacterial activity and tissue repair ability. For diabetes, local persistent inflammation and depression usually lead to the occurrence of non-healing wounds, which greatly increases the substantial burden to patients [143]. Xie and his team developed polyphenol-mediated conductive hydrogel MNs for diabetic wound healing [150]. The MNs were fabricated by using methacrylate gelatin, DA, DA-modified poly (3,4-ethylenedioxythiophene), and lyceum barbarism polysaccharide. Among these MN components, DA, as a common polyphenol, provided long-term anti-inflammatory and antioxidant properties, benefiting the regeneration of wound tissues. Meanwhile, EA stimulation promoted the repair of the peripheral nerve. The proposed MNs provided a new clinical perspective for the treatment of diabetic wounds. The combination of photothermal therapy and MNs is considered an advanced strategy for prompting wound healing [124,151,152]. Due to the excellent chelating ability with metal and high photothermal conversion, polyphenols like PDA and TA can be applied to construct MNs with photothermal therapy capability. In a recently reported study, a bilayer microneedle-based wound dressing was developed for accelerating the healing of diabetic wounds [147]. In the MN system, M2 macrophage-derived exosomes (MEs) were encapsulated in needle tips to exert the anti-inflammatory effect, and PDA-doped polyvinyl alcohol served as the backing layer of the MN patch to produce a mild photothermal effect under 808 nm laser irradiation. The in vitro and in vivo experiments proved that the MNs effectively accelerated diabetic wound healing by suppressing inflammation and promoting angiogenesis, owing to the synergistic effect of MEs and mild photothermal effect (Figure 9A). The MN systems integrated with a sensing unit for monitoring wound conditions have recently gained significant research interest [153]. In the construction of smart MN systems, polyphenols can serve both as bioactive components and as pH indication probes. For example, a multifunctional MNs sensing patch for rapid healing of bacteria-infected wounds and visual monitoring of wound pH was proposed by Zhang et al. [148]. Combining the anti-inflammatory effects of curcumin with the antimicrobial property of metal–organic framework (MOF) hydrogel, the efficiency of wound healing was greatly improved. The FITC, as a kind of pH-sensitive fluorescent indicator, was integrated into MNs substrates to real-time monitor the wound pH based on the changes in fluorescence intensity. Moreover, the detection accuracy of wound pH was improved with the assistance of a machine learning algorithm (Figure 9B).

Although polyphenol-based hydrogel MNs have broad application prospects in the field of wound treatment and diagnosis, some bottlenecks still remain to be broken through. At present, the polyphenol-based hydrogel MNs for wound monitoring are still in the early development stage, and mainly depend on single indicator analysis. Various biochemical signals, including inflammatory factors, enzymes, uric acid, glucose, and pH, are closely correlated to the wound healing phase. In the future, polyphenol-based hydrogel MNs integrated with electrochemical, optical, and other sensing technologies are waiting to be developed to achieve the multi-indicator detection and comprehensive assessment of wound conditions. To reduce the cost and difficulty in design, the current fabrication methods for MNs with dual functions of controlled drug delivery and real-time monitoring should be improved. In addition, flexible wearable electronic devices with a highly sensitive and anti-interference sensing interface should be developed in MNs.

## 5. Conclusions and Perspectives

Various wounds and consequent bacterial infections pose significant challenges to the healthcare system. The early diagnosis, real-time monitoring, and timely treatment hold paramount significance for wound healing and management. In this review, we discuss the current research progress about polyphenol-based hydrogels, mainly focusing on the construction strategy, the applications, and the polyphenol-based hydrogel MNs. The three types of common polyphenol hydrogels, including the TA-based hydrogels, PDA-based hydrogels, and GA-based hydrogels, have been introduced in detail, followed by their construction methods, characteristics, and applications. Owing to their multiple biological activities, such as antibacterial activity, adhesion, and hemostasis, polyphenol-based hydrogels possess great potential in wound treatment.

More importantly, the multifunctionality of polyphenols provides a versatile platform for polyphenol-based hydrogels, enabling them not only to integrate various sensing elements (such as electrochemical and optical elements), but also to advance towards the development of the next generation of intelligent sensing systems. It requires specific design strategies such as incorporating nanomaterials and molecules to improve its conductivity and biocompatibility. The excellent biocompatibility and robust adhesion allow these hydrogel sensors to form intimate interfaces with the skin or internal tissues, enabling the continuous monitoring of a series of physiological biomarkers (such as pH, glucose, lactate, uric acid, and reactive oxygen species) crucial for comprehensive health diagnostics. Moreover, integration with wireless technology (e.g., NFC/Bluetooth) will enable these multiplexed sensing hydrogels to wirelessly transmit data, thereby forming intelligent closed-loop systems for real-time monitoring of dynamic physiological states in wearable and implantable devices [154].

In recent years, MNs have attracted widespread attention in the biomedical field due to their noninvasiveness, simple operation, and effective way for drug delivery without touching blood capillaries and nerve endings. Polyphenol-based hydrogels can serve as the ideal material to fabricate MNs with improved adhesion, good biocompatibility, and antibacterial ability. The recent development of polyphenol-based hydrogel MNs for wound healing and monitoring is discussed in this review. Based on the above summaries and analyses, it is believed that polyphenol-based hydrogels can be built into an intelligent platform integrated with “treatment, monitoring, and response”, showing a wide application prospect in the future.

Although great significant progress has been made for polyphenol-based hydrogels in the field of wound treatment and monitoring, there are still some challenges that need to be solved: (1) More than 8000 types of polyphenols have been identified so far, but only a few have been used to construct polyphenol-based hydrogels, such as TA, PDA, curcumin, and anthocyanin. More kinds of other polyphenols from plants or industries need to be explored to construct hydrogels with potential biological application value. (2) Polyphenol-based hydrogels often exhibit weak cohesion, and their phenolic hydroxyl structures are unstable and susceptible to factors such as temperature, humidity, light, and metal ions, which results in poor long-term stability and diminished bioactivity. Meanwhile, the metabolic degradation, inter-organ translocation, and accumulation of polyphenol-based nanomaterials in the human body have not been fully studied, and the in vivo safety evaluation system remains to be improved. (3) Polyphenol-based hydrogels for wound monitoring usually depend on the single indicator detection, such as pH, which fails to reflect the complicated wound environment for precision management. During the healing process of a wound, chemical and physical indicators involving pH, ROS, protein, enzyme, and glucose are closely related to the wound states. Therefore, it is urgent to develop polyphenol-based hydrogels integrated with multi-mode sensing modules to achieve the simultaneous detection of multiple biomarkers. (4) Polyphenol-based hydrogels for wound treatment and monitoring are a newly developed area. Most of the research focused on the preparation in a laboratory and the verification of the functions. How to scale up the material production and control the quality is another critical issue to be considered for applications.

In terms of clinical applications, a large number of hydrogels have been applied in the biomedical field, including drug delivery, wound dressings, tissue engineering, contact lenses, fillers, etc. However, the components of hydrogels are limited to substances that are widely available, cost-effective, stable in nature, and well-studied, such as common natural polymers (alginate, collagen, etc.) and simple synthetic polymers (polyacrylic acid, polyvinyl alcohol, etc.). Compared with them, polyphenol-based hydrogels have unique advantages, such as the ability to simultaneously meet the requirements of adhesion, toughness, strength, and self-healing. However, they need further improvement before clinical translation, including instability of the oxidized structure during storage and transportation, the difficulty in achieving large-scale production of some complex polyphenolic systems, the increased cost due to the scarcity of some polyphenol sources, and the incomplete toxicological investigation.

To sum up, polyphenol-based hydrogels represent a highly promising research frontier. However, the field is still in its early stages, and significant further investigation is required. This review aims to offer valuable insights for researchers and contribute to accelerating the development of polyphenol-based hydrogel materials.

## Data Availability

No new data were created or analyzed in this study.
